# Basketball-Specific Small-Sided Games Training during Ramadan Intermitting Fasting: Do Changes in Body Composition, Sleep Habits, and Perceived Exertion Affect Technical Performance?

**DOI:** 10.3390/ijerph182212008

**Published:** 2021-11-16

**Authors:** Seifeddine Brini, Daniel Castillo, Javier Raya-González, Carlo Castagna, Anissa Bouassida, Riadh Khalifa, Sabri Gaied Chortane, Filipe Manuel Clemente

**Affiliations:** 1 Research Unit, Sportive Performance and Physical Rehabilitation, High Institute of Sports and Physical Education of Kef, University of Jendouba, Kef 7100, Tunisia; bseifeddine15@gmail.com (S.B.); bouassida_anissa@yahoo.fr (A.B.); 2 Faculty of Education, Universidad de Valladolid, 42004 Soria, Spain; danicasti5@gmail.com; 3 Faculty of Health Sciences, University Isabel I, 09003 Burgos, Spain; rayagonzalezjavier@gmail.com; 4 School of Sports and Exercise Science, University of Rome Tor Vergata, 00118 Rome, Italy; castagnac@libero.it; 5 Fitness Training and Biomechanics Laboratory, Italian Football Federation (FIGC), Technical Department, 50135 Florence, Italy; 6 Higher Institute of Sport and Physical Education of Ksar-Said, University of La Manouba, Tunis 2010, Tunisia; riadhkhal@yahoo.fr; 7 Laboratory of Cardio-Circulatory, Respiratory, Metabolic and Hormonal Adaptations to Muscular Exercise, Faculty of Medicine Ibn El Jazzar, Sousse 4002, Tunisia; Sabrigaied1@gmail.com; 8 Escola Superior Desporto e Lazer, Instituto Politécnico de Viana do Castelo, Rua Escola Industrial e Comercial de Nun’Álvares, 4900-347 Viana do Castelo, Portugal; 9 Instituto de Telecomunicações, Delegação da Covilhã, 1049-001 Lisboa, Portugal

**Keywords:** team sport, performance, fatigue, intermitting fasting, nutrition

## Abstract

The objective of this study was to assess the effects of an additional small-sided games (SSGs) training program during Ramadan intermitting fasting (RIF) on technical performance depending on changes in body composition, sleep habits, and ratings of perceived exertion (RPE). Twenty-four professional male basketball players from the Tunisian first division participated in this study. The players were randomly assigned to an intervention group (INT; *n* = 12) or an active control group (CON; *n* = 12). Both groups completed a four-week SSG training program (three sessions per week). During the first and fourth weeks of the SSGs training, the two groups were evaluated to detect changes in technical performance, dietary intake, body composition, sleep quality index (PSQI) survey outcomes, RPE, heart rate (HR), and blood lactate concentration [La]. During the fourth week of the RIF period, body composition, dietary intake, sleep latency, sleep duration, and HR significantly decreased only for INT (*p* < 0.001). However, RPE significantly increased (*p* < 0.001), and technical performances were negatively affected (*p* < 0.01). MANCOVA (adjusted for the percentage of change in sleep duration, body mass, and RPE) showed no significant differences in either group. In conclusion, our results showed that the technical performance of professional basketball male players was significantly affected at the end of RIF independently of changes in RPE, sleep habits, and body composition.

## 1. Introduction

Small-sided games (SSGs) are small and adjusted versions of formal matches intended to introduce specific tactical aspects of the game while changing task constraints to promote variations in the acute physiological and physical responses of players [1,2,3]. These drill-based games involve fewer players per team than in official matches, are played in reduced spaces, and apply modified rules depending on the team’s goals while maintaining the game’s principles [4,5]. Additionally, SSGs are very popular training drills for basketball players of all abilities and competitive levels, and they constitute an alternative to traditional fitness training [6,7,8,9]. In addition, the main benefit of SSGs is that they develop or maintain players’ technical skills, which is particularly important in basketball [10,11,12].

Several factors potentially affect and may contribute to a differentiation in the frequencies of technical actions performed by players during basketball SSGs. For example, previous studies revealed that the smaller format of play increased the execution of most technical actions (i.e., dribbling, passing, shooting, and rebounds) [6,9,12]. Moreover, the anthropometric and fitness profiles of players (e.g., body height, body mass, neuromuscular capacity, aerobic fitness) can affect the capacity of players to perform specific actions [10]. Similarly, Clemente et al. [13] reported an association between players’ anthropometric and fitness characteristics and technical demands during basketball SSGs. Moreover, Sampaio et al. [14] showed that the tallest and heaviest players succeeded more often in using their body size as an advantage. Furthermore, Ibáñez et al. [15] reported that forwards and centers are the tallest and heaviest players, and they commit fewer turnovers than guards, who tend to be smaller and lighter. Therefore, considering the possible simultaneous beneficial effects of SSGs on players’ technical skills and anthropometric parameters (e.g., body mass, body mass index, and body fat percentage level) may be important to assessing the association between these two variables.

Previous studies reported that body composition may be influenced by several factors such as daily nutritional habits, training status, and climatic and environmental issues [16,17]. In the particular case of the Muslim culture, factors such as Ramadan intermittent fasting (RIF) can also lead to changes in body composition [16,17,18]. In fact, RIF is characterized by dietary habits that are not similar to those in other periods. Thus, the amounts of fat, protein, and carbohydrates consumed may vary during this month and influence the body composition of Muslim athletes [16,19].

In addition, chronological, physiological, metabolic, and hormonal changes occur during RIF, making it a unique model for prolonged intermittent fasting’s effects on athletic performance [20]. In this context, Brini et al. [17] showed that SSG training during RIF leads to a significant decrease in body composition in male senior basketball players. Moreover, RIF is characterized by habit changes (e.g., delayed bedtime, shortened sleep duration, and partial sleep deprivation) [21]. In this context, previous studies reported the importance of sleep and its impact on physical and mental wellbeing [22]. A lack of sleep results in fatigue and sleepiness, and decreases in alertness, vigilance, working memory, concentration, and problem-solving ability [22].

To the best of our knowledge, no previous study has investigated the potential effects of changes in body composition and sleep habits on technical performance among basketball players during RIF. However, this topic may be important for coaches to properly manage training sessions and avoid the negative effect of these parameters. Therefore, the aim of this study was to assess the effect of including an additional SSG training program during RIF on technical performance by considering changes in body composition, sleep habits, and ratings of perceived exertion. Considering the previous literature [11,13,17,21], we hypothesized that changes in body composition, sleep habits, and perceived exertion will negatively affect technical performance during SSGs.

## 2. Materials and Methods

### 2.1. Study Design and Setting

**Figure 1:**
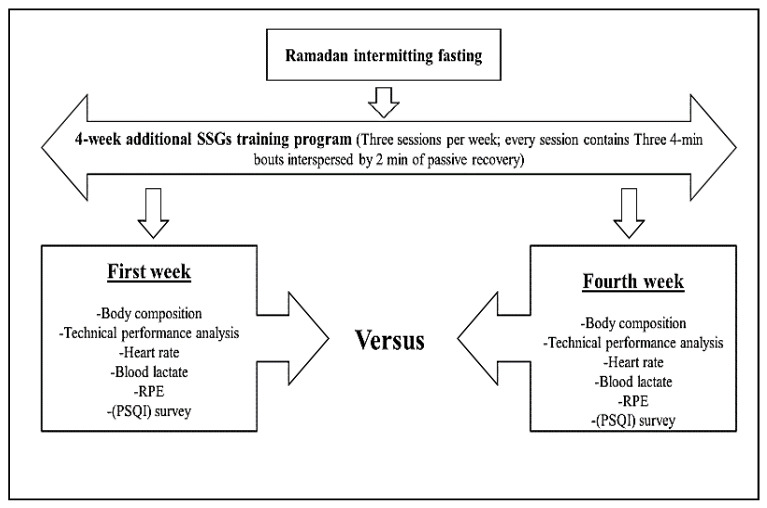
Experimental design.

A pre-post crossover study design was used to conduct the experiment. Subjects were divided into two subgroups, with twelve players in each. Overall, the study lasted 5 weeks and was conducted during the 2018/2019 season. The experimental period started with the beginning of the RIF in May 2019 and lasted until June 2019. The length of each fasting day was approximately 16–17 h. (temperature: 27.2 ± 3.7 °C; relative humidity: 62.1 ± 11.4%). During the RIF period, participants exercised five times per week between 5:00 and 6:30 p.m. and completed one game per week on the weekend. Participants completed a 4-week SSG training with a frequency of three sessions per week (Monday, Wednesday, and Friday) over the course of the study period. No additional exercises of strength and conditioning were conducted by any of the experimental groups. During the first and the fourth weeks of the SSG training, the two groups were evaluated to detect changes in technical performance, dietary intake, body composition, sleep quality index (PSQI) survey outcomes, rating of perceived exertion scale (RPE), heart rate (HR) and blood lactate concentration [La] (Figure 1).

### 2.2. Participants

Twenty-four professional basketball players from two different teams of the Tunisian first division volunteered to participate in this study (Table 1). During the experimental period the two teams were of the same level and practically had the same ranking in the championship. For both teams, the players had similar training experience (12.32 ± 1.29 years) and weekly practice load (≈9 h). An a priori power calculation showed a required minimal sample size of 12 for a power of 0.80 (G*Power v. 3.0). Prior to the start of the study, subjects undertook several tests to determine their baseline physical fitness and technical skill levels because it is well-established that basketball technical and physical skills are highly dependent on players’ roles on the court [23]. We did not record a significant difference between groups in baseline tests, and the randomization process took this into account. Players in each team were matched according to their playing position. Each group included 3 guards, 3 shooting guards, 2 small forwards, 2 forwards, and 2 centers. Random allocation within each pair to either an intervention group (INT; *n* = 12) or an active control group that was not fasting during the RIF (CON; *n* = 12) was then performed by tossing a coin. In addition, 50% of the total number of participants were committed to fasting. The inclusion criteria for study participation were: (1) participation in at least 90% of the training sessions; (2) Muslims who were fasting during the RIF (only for the INT group); (3) having good health (no pain or injury reported); (4) and not taking any medications or other drugs. The study was conducted during the competitive season and was approved by a local Clinical Research Ethics Committee (approval No. 8/2018), and the protocol was conducted according to the Declaration of Helsinki. All participants provided their written informed consent to participate in the study.

### 2.3. Procedures

Players were familiarized with all procedures before the start of the experimental protocol. To minimize any effects of diurnal variations the training sessions were conducted at the same time of day. Players were instructed to wear the same footwear during all test sessions.

In both groups, training sessions started with a 15 min warm-up program consisting of 5 min of low-intensity running, 5 min of dynamic stretching, and 5 min of skipping exercises, followed by technical and tactical drills based on basic basketball movements (i.e., offensive, ready stance, running, change in direction, linear sprint, stopping, pivoting, and jumping exercises), specific basketball movements (triple threat position, pivot, face up or one- and two-phase stop), basketball technique fundamentals (dribbling, passing, and shooting), and basic defensive movements (defensive stance, defensive slide, denial defense, and box-out). Both groups completed the same training volume (~90 min per session) over the course of the study (Table 2).

### 2.4. Small Sided Games Training Program

Small-sided games were performed on half-court (14 × 15 m) (35 m^2^ per player) at the beginning of the training session. The total duration of each SSG was 12 min. The only defensive strategy allowed was man-to-man. The training regimes consisted of three 4 min bouts interspersed by 2 min of passive recovery. The court size and the duration of (3 vs. 3) SSGs were strictly controlled as reported in previous studies [9,11]. In each group (INT or CON), players were allocated to four balanced teams (three players in each team). Throughout the experimental period, teams from INT played only against each other and teams from CON played only against each other. These choices were used to determine the effects of the RIF. Moreover, the players were asked to perform at maximum effort during the games. Two coaches were positioned around the perimeter of the court to encourage the players and to provide new balls when necessary to allow for continuous play and to maintain the game pace during the SSG sessions.

#### Rules of the Small Sided Game

New ball possession: (Basket made, foul, rebound offensive or defensive, steal, turnover, out of bounds). Ball clearance: (pass to assistant except after offensive rebound). Shot clock: (12 s). Free throws: (1 point to offensive team). Referees: (two).

### 2.5. Training Load Monitoring

To determine whether the subjects’ global training load remained consistent through the study, the session rating of perceived exertion (RPE) training score was taken following each session. About 30 min after the training sessions, subjects were asked to rate the global intensity of the entire workout session using the category ratio-10 RPE scale according to the methods described by [24]. A daily training load was created by multiplying the training duration (minutes) by the session RPE. The weekly training load was determined by summing the daily training loads for each athlete during each week.

### 2.6. Assessment

#### 2.6.1. Anthropometrics

The body mass was evaluated in kilograms with a precision of 0.1 kg using an electronic balance (Pharo 200 Analytic, Germany) which was calibrated regularly. It was recommended to be presented without shoes and with light clothes. Height was measured in centimeters with a precision of 0.1 cm using a portable stadiometer (Seca, Maresten, UK). The body mass index (BMI) was then determined by dividing the weight (kg) by the square of the height (m). The body fat percentage was determined using the four skin folds method (biceps, triceps, subscapular, and suprailiac skin folds) using pliers (Harpenden caliper). The fat percentage: BF% = (4.95/Body density − 4.5) × 100; (Body density = 1.162 2 0.063 3 log the sum of four skin folds) [25]. Anthropometric measurements were taken according to the recommendations from the International Society for the Advancement of Kinanthropometry (ISAK). All these measures were taken by a qualified technician (ISAK level 3).

#### 2.6.2. Dietary Intake

Participants noted all meals consumed throughout the experimental period, recording both the amounts and types of food and fluid consumed, and they were also interviewed and analyzed by a nutritionist. Findings were analyzed using the software program Bilnut (Nutrisoft Bilnut: Food Survey Program version 2.01) and the food-composition tables of the Tunisian National Institute of Statistics (1978).

#### 2.6.3. The Pittsburgh Sleep Quality Index

The validated Arabic version of the Pittsburgh Sleep Quality Index [26] assessed subjective sleep quality during the previous month [27]. It comprises 19 questions, covering seven components of sleep: duration, quality, latency, efficiency, disturbances, daytime dysfunction, and the use of sleeping medications. The total score ranges from 0 to 21, where “0” designates no troubles and “21” indicates severe problems in all areas of sleep.

#### 2.6.4. Physiological Measures

Heart rate was continuously monitored throughout the training intervention by HR monitors (Polar Team Sport System; Polar-Electro OY, Kempele, Finland) and was recorded at a 5 s interval. To reduce HR recording error, all players were regularly asked to check their HR monitors during the SSG. Heart rate data were expressed both as absolute HR and percentage of HRmax (%HRmax). The HR values were relativized by the HRmax presented by each player in the Yo-YoIR1. The %HRmax was calculated using the following formula: %HRmax = (HRmean/HRmax) × 100. Blood samples for the determination of blood lactate concentration [La] were collected 3 min after training in the absence of active recovery [28]. Blood samples, taken from the fingertip of the index finger, were analyzed by a validated portable analyzer (Lactate Pro, Arkray, Japan).

#### 2.6.5. Video Analysis

Technical demands were assessed using a notational analysis. The SSGs were recorded using 2 cameras (HDR-CX450, Sony, Japan) placed at the right half-court corner and elevated about 3.5 m. Videos were subsequently analyzed throughout each SSGs to evaluate the following technical parameters: Individual (Dribbles, Passes, Shots, Blocks, Rebounds, Fouls, Steals); and Team (Turnovers, Ball possessions, Recovered balls, Ball reversals, Dribbles in key area, Screens, Hand-offs) [11]. In order to verify the reliability of the measures, the videos were scored and rechecked after one week, resulting in the following: intraclass correlation coefficient = 0.91; 95% confidence interval: 0.72–0.99; coefficient of variation: 4%.

### 2.7. Statistical Analyses

Data are presented as means and standard deviations (SD). After normality of data was tested and confirmed using the Shapiro–Wilk test, baselines between group differences were computed using t-tests for independent samples. The ICCs were used to check reliability of the video score. Firstly, to investigate the variation (first week to fourth week) of body composition, sleep habits, RPE, and technical performance, a 2 (groups: INT, CON) × 2 (time: week1, week4) repeated measures ANOVA was performed. If a statistically significant interaction effect was found, Bonferroni corrected post-hoc tests were calculated. Additionally, effect sizes (ES) were determined from ANOVA output by converting partial eta-squared to Cohen’s d [29]. Following Hopkins et al. [30], ES were considered trivial (<0.2), small (0.2–0.6), moderate (0.6–1.2), large (1.2–2.0), and very large (2.0–4.0). Secondly, to document the influence of body composition, sleep habits, and RPE at the end of the experimental period on technical performance, three separate multivariate analyses of covariance (MANCOVAs) adjusted for percentage of change (first week to fourth week) in sleep duration, body mass, and RPE, separately. For MANCOVAs, F-values, *p*-values, and effect sizes (η^2^) were reported. Pearson’s linear correlation coefficient was used to check the relationship between the anthropometric and technical profile. The magnitude of the correlation was expressed as trivial: r < 0.1; low: 0.1–0.3; moderate: 0.3–0.5; large: 0.5–0.7; very large: 0.7–0.9; nearly perfect > 0.9; and perfect: 1. The level of significance was set at *p* < 0.05. All statistical analyses were computed using SPSS for Windows, version 20.0 (SPSS Inc., Chicago, IL, USA).

## 3. Results

No injuries occurred over the course of the study. During the four-week intervention period, adherence rates were 97.6% for INT and 97.2% for CON. The average playing time per game was 29.6 ± 1.7 min for INT and 29.1 ± 1.6 min for CON. No statistically significant between-group differences were observed for these measures.

- Body composition

Bonferroni corrected post hoc tests revealed a significant decrease in BM, BMI, and BF% at the end of the experimental period only for INT (*p* < 0.001) (Table 3).

- Dietary intake

Bonferroni corrected post hoc tests revealed a significant decrease in total energy intake, carbohydrate intake, protein intake, and fat intake at the end of the experimental period only for INT (*p* < 0.001) (Table 3).

- Sleep patterns

Bonferroni corrected post hoc tests revealed a significant decrease in sleep latency, sleep duration, and total score of PSQI, and a significant increase in sleep efficiency at the end of the experimental period only for INT (*p* < 0.01) (Table 4).

- RPE

Bonferroni corrected post hoc tests revealed a significant increase in RPE at the end of the experimental period only for INT (*p* < 0.001) (Table 5).

- Physiological Measures

Bonferroni corrected post hoc tests revealed a significant decrease in absolute HR and percentage of HRmax during the fourth week of the RIF only for INT (*p* < 0.001) (Table 5). Blood lactate did not change significantly during the fourth week of the RIF in comparison with the first week for INT.

- Individual and Team Technical Parameters

Bonferroni corrected post hoc tests revealed a significant decrease in dribbles, 2pts%, 3pts%, and steals, and a significant increase in passes, total shots, and fouls at the end of the experimental period only for INT (*p* < 0.01). Moreover, a significant increase was recorded in turnovers, recovered ball, ball reversals, screens off, screens on, and hand-offs, and a significant decrease in dribbles in key area (*p* < 0.001) (Table 6).

- MANCOVA results

The three separate multivariate analyses of covariance (MANCOVA) adjusted for percentage of change (first week to fourth week) in sleep duration, body mass, and RPE, separately, showed that Wilks’ λ in both groups were not significant (Table 7).

- Correlations analysis between individual technical parameters and other variables

During the fourth week of the RIF, correlation analysis showed that dribbles was positively correlated with BF% (r = 0.62, *p* = 0.03, large magnitude); 2pts% was positively correlated with absolute HR (r = 0.59, *p* = 0.04, large magnitude); 3pts% was negatively correlated with sleep latency (r = −0.79, *p* = 0.002, large magnitude); steels was negatively correlated with sleep latency (r = −0.21, *p* = 0.05, low magnitude); and total shots was positively correlated with sleep efficiency (r = 0.68, *p* = 0.016, large magnitude).

- Correlations analysis between team technical parameters and other variables

During the fourth week of the RIF, correlation analysis showed that dribbles in key area was positively correlated with [La] (r = 0.73, *p* = 0.026, very large magnitude); and negatively correlated with RPE (r = −0.75, *p* = 0.021, very large magnitude) and sleep duration (r = −0.80, *p* = 0.010, very large magnitude). Moreover, ball possession was positively correlated with sleep latency (r = −0.79, *p* = 0.011, very large magnitude).

## 4. Discussion

This study investigated the effects of introducing an additional SSG training program during RIF on technical performance based on changes in body composition, sleep habits, and RPE. The main results indicated a significant decrease in body composition during the fourth week of RIF, which was associated with a significant change in sleep habits and an increase in RPE for INT. Moreover, individual and team technical performances were negatively affected during the fourth week of RIF for INT, independently of changes in body composition, sleep habits, and RPE, which conflicted with our hypothesis. Otherwise, players maintained a high training load (2000 AU for INT) in order to maintain their competitive fitness and muscle mass during the experimental period [31,32]. The weekly training load measured in this study was similar to training loads reported during the in-season period in Italian soccer players (1900 AU) but lower than those observed in overreached rugby league players [19,33].

In the present study, the significant decrease in body composition of INT recorded at the end of the RIF period may be attributed to the significant decrease in dietary intake during the fourth week of RIF for INT. In fact, our findings showed that total calorie intake was significantly decreased during the fourth week compared to the first week of RIF in the CON group. Our findings were consistent with previous studies indicating a decrease in body composition during RIF in athletes in general, and basketball players specifically [16,17]. It has been suggested that the observed decline in body composition is attributed to a decrease in fluid intake and hypohydration with little loss of body fat during RIF [16,17,19]. These findings are important because RIF is an integral part of the Muslim religion and may have various implications for energy requirements, and mixed results have been reported in this regard [16,17,19,34,35]. Previous studies reported that RIF is characterized by alterations in meal schedules and frequency—specifically, meals are eaten only at night and are eaten less frequently than usual. Hence, this may affect energy and nutrient intake [36]. In contrast, the findings of the present study do not support some previous research that showed no changes in body mass or dietary intake among athletes during RIF [34,35,37]. Those results were explained by the common belief that athletes are likely to overcompensate for their reduced food and fluid intakes during RIF, especially during Sahur [38,39].

Coaches and players recognize that sleep is a crucial component of successful training, competition, and recovery [22]. The importance of sleep for athletic performance has been presented in the literature [22]. However, sleep among athletes remains poorly described, especially during RIF, when significant diurnal changes are known to occur [36]. In fact, individuals tend to prepare for RIF by waking early and eating a meal before sunrise [36]. As a result, athletes likely suffer from sleep loss or sleep fragmentation, which may reduce athletic performance [36]. This line of reasoning is in accordance with the results of the present study. Moreover, our findings were similar to those of previous studies indicating the negative effect of RIF on subjective sleep quality [40,41].

Moreover, Roky et al. [21] reported that if loss of sleep accumulates during RIF, it could lead to a disruption of the sleep–wake cycle, thereby inducing more fatigue and reducing mental and physical performance. Our results support these findings concerning the increase in fatigue. We recorded a higher RPE value at the end of the RIF period than during the first week for INT. The INT group’s RPE was also higher than the CON group’s RPE during the same week. Our findings may be explained by the increase in muscle fatigue during the end of RIF, which leads to an increase in perceived exertion. This explanation is concordant with previous research that reported increased fatigue [42].

The increased RPE score suggests that RIF results in an increased level of fatigue during training and, more concerningly, increased incidences of injury and illness. Indeed, decreases in physical function can increase perceived exertion and lead to an earlier onset of fatigue, and, thus, an increased risk of injury or illness [43]. Otherwise, blood lactate concentration did not change significantly throughout RIF for the INT group. These results indicate that the participants maintained similar metabolic conditions for each SSG session. Concerning HR, the present study showed that this parameter significantly decreased at the end of RIF in comparison with the first week only for the INT group. Accordingly, Al Suwaidi et al. [44] explained this decrease by fasting’s associations with catecholamine inhibition and reduced venous return, which decreases the sympathetic tone, ultimately leading to a decrease in blood pressure, HR, and cardiac output.

Finally, the technical performance of INT was adversely affected at the end of RIF in comparison with CON. Our findings were mainly related to the sensation of fatigue recorded during the fourth week of RIF. In fact, the present study reported a significant correlation at the end of the RIF period between RPE, some sleep parameters, and technical performance for INT. This may, in part, explain the negative findings concerning players’ technical profiles at the end of the RIF period. Moreover, the level of fatigue recorded during the fourth week of RIF may also be related to low carbohydrate intake during Ramadan. Indeed, a low-carbohydrate diet can reduce the buffering capacity of the blood during intense muscular contractions [45]. Therefore, if the carbohydrate intake significantly decreases at the end of RIF, fasting may have adverse effects on muscle power.

Additionally, significant decreases in individual actions, such as dribbles, blocks, and steals, alongside the increased numbers of mid-range and three-point shot attempts, influenced the collective aspect and the game mode applied to SSGs. During the first week of RIF, the collective technical profile was based on relatively high numbers of ball possessions, dribbles in the key, drives with lay-ups, and handoffs. However, the fourth week of RIF was characterized by significantly fewer ball possessions, handoffs, and dribbles in the key, together with significantly more mid- and long-range shot attempts.

The accumulated fatigue recorded during the fourth week of RIF influenced the progress of SSGs and the strategies adopted by players during attack phases. These influences are likely based on one vs. one duels during the first week of RIF (due to the fast game rhythm) and the high numbers of mid- and long-range shots during the fourth week (due to a slow game rhythm). Moreover, the defensive aspect of the game appears to be negatively affected by RIF, as indicated by the lower numbers of blocks and steals.

### Limitations

Although we present a novel addition to the literature, our study has some limitations that warrant consideration. First, the small sample size reduces the results’ statistical power. Second, time-motion variables (i.e., distance covered and frequency of sprints and high-intensity runs) were not included, which would have provided an additional level of fidelity to our study. Third, only one SSG format (3 vs. 3) was used—changing parameters such as the duration, court size, recovery period, and number of players may provide further information or yield different results. Finally, the period within the year when Ramadan occurred must be considered. Future studies may produce different results by investigating the month of Ramadan under different climatic circumstances.

## 5. Conclusions

Our results showed that the technical performance of professional male basketball players was significantly affected at the end of RIF independently of changes in RPE, sleep habits, and body composition. Moreover, HR significantly decreased during the fourth week of RIF. The results should be considered by researchers and practitioners when programming training regimens during RIF.

## Figures and Tables

**Table 1:** Characteristics of basketball players.

Basketball Players	Age (Years)	Height (cm)	Body Mass (kg)	BMI (kg·m^−2^)	VO_2max_ (mL∙min^−1^∙kg^−1^)
INT (*n* = 12)	25.32 ± 2.56	1.96 ± 0.07	88.08 ± 6.38	23.43 ± 1.19	51.10 ± 2.1
CON (*n* = 12)	24.85 ± 1.55	1.93 ± 0.09	88.17 ± 7.27	23.41 ± 1.46	50.80 ± 2.4

Legend: Data are means and standard deviations. INT: intervention group; CON: control group; BM: body mass; BMI: body mass index; VO_2max_: Maximal oxygen consumption.

**Table 2:** Weekly training program during the month of Ramadan for intervention and control groups.

Days	Weekly Training Program during the Month of Ramadan for INT and CON Groups
Monday	-Warm up, 15 min -Small sided games, (3 × 4 min), 2 min rec between bouts -Free throw shooting, 10 min -Technical training, 20 min -Tactical training, 20 min
Tuesday	-Warm up, 15 min -Specific basketball fundamental training, 15 min -moderate intensity mid-range and 3 pts shot exercise, 20 min - Technical/Tactical training, 40 min
Wednesday	-Warm up, 15 min -Small sided games, (3 × 4 min), 2 min rec between bouts -Free throw shooting, 10 min -Technical training, 20 min -Tactical training, 20 min
Thursday	-Warm up, 15 min -Specific basketball fundamental training, 15 min -Moderate intensity ball drill transition training, 16 min -moderate intensity mid -range and 3pts shot exercise, 20 min -Tactical training, 20 min
Friday	-Warm up, 15 min Small sided games, (3 × 4 min), 2 min rec between bouts -Free throw shooting, 15 min -low intensity 3pts shooting exercises, 25 min
Saturday	-Match
Sunday	-Recovery

**Table 3:** Body composition and estimated daily dietary intake recorded during the first and the fourth weeks of the experimental period for the intervention and the control groups.

Variables		First Week		Fourth Week		*p* Value (ES)		
		INT	CON	INT	CON	Time	Group	Group × Time
BM		88.08 ± 6.38	88.17 ± 7.27	84.83 ± 6.24	88.08 ± 7.11	0.000 (0.81)	0.537 (0.03)	0.000 (0.84)
BMI		23.43 ± 1.19	23.41 ± 1.46	23.34 ± 1.18	23.40 ± 1.49	0.000 (0.84)	0.976 (0.001)	0.001 (0.66)
BF%		12 ± 2.41	12.16 ± 2.69	10.96 ± 2.21	12.21 ± 2.73	0.003(0.56)	0.286 (011)	0.002 (0.59)
Total energy intake		2741.66 ± 172.98	2829.17 ± 73.04	2183.33 ± 174.95	2763.33 ± 95.19	0.000 (0.80)	0.000 (0.91)	0.000 (0.73)
Carbohydrate intake	(g)	307.50 ± 19.13	309.16 ± 17.82	284.17 ± 16.21	302.50 ± 12.15	0.022 (0.39)	0.084 (0.25)	0.004 (0.54)
	(%)	45.01 ± 3.76	43.74 ± 2.73	52.40 ± 5.51	43.84 ± 2.41	0.010 (0.47)	0.001 (0.65)	0.005 (0.52)
Protein intake	(g)	88.33 ± 5.02	84 ± 4.77	83.33 ± 4.68	85.08 ± 4.46	0.001 (0.68)	0.493 (0.04)	0.000 (0.84)
	(%)	12.94 ± 1.13	11.88 ± 0.67	15.33 ± 1.30	12.32 ± 0.62	0.000 (0.74)	0.000 (0.83)	0.007 (0.50)
Fat intake	(g)	121.66 ± 11.74	123.33 ± 9.61	96.25 ± 7.42	127.50 ± 11.96	0.000 (0.74)	0.000 (0.80)	0.000 (0.83)
	(%)	40.10 ± 4.67	39.22 ± 2.72	39.85 ± 3.75	41.50 ± 3.25	0.342 (0.08)	0.636 (0.02)	0.272 (0.11)

Legend. Data are means and standard deviations. INT: intervention group; CON: control group; BM: body mass; BMI: body mass index; BF%: Body fat percentage.

**Table 4:** Measurement of the subjective quality of sleep recorded during the first and the fourth weeks of the experimental period for the intervention and the control groups.

Variables	First Week		Fourth Week		*p* Value (ES)		
	INT	CON	INT	CON	Time	Group	Group × Time
Sleep latency (min)	14.50 ± 1.17	15.25 ± 0.75	12.08 ± 0.90	14.83 ± 0.72	0.000 (0.77)	0.000 (0.85)	0.001 (0.65)
Sleep efficiency (%)	93.50 ± 1	94.83 ± 0.97	95 ± 0.94	95.42 ± 0.80	0.000 (0.73)	0.004 (0.55)	0.042 (0.33)
Sleep duration (h)	9.83 ± 1.64	9.42 ± 1.51	7 ± 0.74	9.58 ± 1.24	0.002 (0.60)	0.007 (0.50)	0.000 (0.83)
Total score of PSQI	4.83 ± 0.72	4.75 ± 0.87	3.75 ± 0.75	4.92 ± 0.67	0.059 (0.29)	0.078 (0.26)	0.004 (0.54)

Legend. Data are means and standard deviations. INT: intervention group; CON: control group; PSQI: Pittsburgh Sleep Quality Index.

**Table 5:** Measurement of heart rate, blood lactate, and rating of perceived exertion recorded during the first and the fourth weeks of the experimental period for the intervention and the control groups.

Variables	First Week		Fourth Week		*p* Value (ES)		
	INT	CON	INT	CON	Time	Group	Group × Time
HRmean (beat·min^−1^)	187.65 ± 1.79	187.80 ± 1.91	183.55 ± 1.05	187.97 ± 2.18	0.000 (0.87)	0.000 (0.71)	0.000 (0.86)
%HRmax (beat·min^−1^)	92.25 ± 0.90	92.18 ± 1.03	90.24 ± 0.75	92.27 ± 1.01	0.000 (0.88)	0.017 (0.42)	0.000 (0.86)
[La] (mmol·l^−1^)	8.23 ± 1.90	7.84 ± 2.36	8.24 ± 1.97	7.87 ± 2.38	0.263 (0.11)	0.329 (0.09)	0.901 (0.001)
RPE	6.58 ± 0.51	6.25 ± 0.75	7.67 ± 0.49	6.50 ± 0.52	0.005 (0.53)	0.001 (0.68)	0.005 (0.52)

Legend. Data are means and standard deviations. INT: intervention group; CON: control group; HRmean: heart rate mean; %HRmax: percentage of heart rate max; [La]: blood lactate; RPE: rating of perceived exertion.

**Table 6:** Variations of individual and team technical parameters during the experimental period for the intervention and the control groups.

Variables		First Week		Fourth Week		*p* Value (ES)		
		INT	CON	INT	CON	Time	Group	Group × Time
INDIVIDUAL								
Dribbles		27.50 ± 7.38	26.58 ± 6.65	21.50 ± 5.14	24.42 ± 5.80	0.001 (0.65)	0.093 (0.24)	0.002 (0.61)
Passes		20.75 ± 6.35	23.25 ± 5.15	25 ± 4.81	21.67 ± 5.77	0.009 (0.47)	0.384 (0.07)	0.003 (0.58)
Shots	Total	15.17 ± 3.13	14.83 ± 2.95	17.83 ± 3.27	15.58 ± 2.64	0.001 (0.64)	0.010 (0.47)	0.038(0.34)
	2pts%	41 ± 12.17	40.25 ± 11.23	38 ± 10.62	39 ± 10.99	0.045 (0.32)	0.773 (0.008)	0.031(0.36)
	3pts%	41.33 ± 7.45	39.92 ± 8.07	36.25 ± 6.93	38.66 ± 7.83	0.003 (0.57)	0.374 (0.07)	0.001(0.67)
Block		1.58 ± 1.31	1.59 ± 1.38	0.58 ± 0.52	0.83 ± 0.58	0.020 (0.41)	0.275 (0.11)	0.274 (0.10)
Rebounds	Offensive	2.17 ± 1.40	1.92 ± 1.38	3 ± 1.47	2.50 ± 1.51	0.040 (0.33)	0.021 (0.40)	0.429(0.06)
	Defensive	6 ± 1.86	6.25 ± 1.60	7.42 ± 1.83	6.92 ± 1.97	0.020 (0.40)	0.339 (0.08)	0.069(0.27)
Fouls		2.08 ± 1.24	2.33 ± 1.07	3.17 ± 0.72	2.67 ± 1.07	0.012 (0.45)	0.429 (0.06)	0.043 (0.32)
Steal		0.83 ± 1.03	0.66 ± 0.89	0.67 ± 0.78	1.08 ± 0.90	0.612 (0.02)	0.389 (0.07)	0.027 (0.37)
TEAM								
Turnovers		7.69 ± 0.85	8.41 ± 0.75	11.58 ± 0.43	9.45 ± 0.88	0.000 (0.86)	0.011 (0.57)	0.000 (0.90)
Ball possessions/min		8.45 ± 0.61	8.43 ± 0.38	5.70 ± 0.09	6.17 ± 0.08	0.000 (0.98)	0.125 (0.27)	0.117 (0.28)
Recovered balls/possession		0.25 ± 0.01	0.20 ± 0.02	0.30 ± 0.01	0.37 ± 0.02	0.000 (0.99)	0.070 (0.35)	0.000 (0.96)
Ball reversals		12.89 ± 0.78	12.22 ± 0.97	15 ± 0.87	13 ± 0.71	0.000 (0.85)	0.005 (0.64)	0.011 (0.57)
Dribbles in key area		42.66 ± 1.32	37.22 ± 1.20	22.89 ± 0.78	26.11 ± 0.78	0.000 (0.99)	0.030 (0.46)	0.000 (0.97)
Screens	Off	12.11 ± 0.78	13.44 ± 0.88	20.22 ± 0.67	15.11 ± 0.78	0.000 (0.98)	0.000 (0.80)	0.000(0.96)
	On	8.33 ± 0.71	9.89 ± 0.78	12.22 ± 0.66	11 ± 0.71	0.000 (0.97)	0.471 (0.07)	0.002(0.73)
Hand-offs		10.44 ± 0.88	10.78 ± 0.67	13.44 ± 0.73	11.67 ± 0.70	0.000 (0.96)	0.069 (0.35)	0.007 (0.62)

Legend. Data are means and standard deviations. INT: intervention group; CON: control group.

**Table 7:** Results of MANCOVA for individual technical performances for the intervention and the control groups.

Variables		Multivariate Tests																							
		Covariate				MANCOVA				Covariate				MANCOVA				Covariate				MANCOVA			
		% SD				(Adjusted for % SD)				% BM				(Adjusted for % BM)				% RPE				(Adjusted for % RPE)			
		Wilks’ λ	F	*p*	*η^2^*	Wilks’ λ	F	*p*	*η^2^*	Wilks’ λ	F	*p*	*η^2^*	Wilks’ λ	F	*p*	*η^2^*	Wilks’ λ	F	*p*	*η^2^*	Wilks’ λ	F	*p*	*η^2^*
INDVIDUAL		0.40	1.64	0.214	0.599	0.73	0.41	0.915	0.271	0.65	0.60	0.789	0.351	0.46	1.29	0.342	0.539	0.34	2.18	0.109	0.664	0.48	1.72	0.397	0.516
		*p*				F	*p*		*η^2^*	*p*				F	*p*		*η^2^*	*p*				F	*p*	*η^2^*	
Dribbles		0.202				1.30	0.27		0.06	0.581				0.97	0.336		0.05	0.279				0.10	0.750	0.005	
Passes		0.457				0.89	0.36		0.04	0.431				0.75	0.395		0.04	0.699				1.63	0.216	0.075	
Shots	Total	0.712				0.009	0.92		0.000	0.863				0.001	0.972		0.000	0.406				0.35	0.475	0.026	
	2pts%	0.119				0.007	0.93		0.000	0.674				1.19	0.287		0.056	0.051				4.16	0.055	0.172	
	3pts%	0.160				0.11	0.74		0.006	0.343				0.26	0.614		0.013	0.368				0.08	0781	0.004	
Block		0.438				0.16	0.69		0.008	0.492				0.002	0.967		0.000	0.015				1.94	0.179	0.088	
Rebounds	Offensive	0.162				0.21	0.65		0.01	0.549				1.38	0.254		0.06	0.458				0.16	0.694	0.008	
	Defensive	0.674				0.75	0.40		0.04	0.297				1.07	0.313		0.05	0.300				2.11	0.162	0.096	
Fouls		0.226				2.59	0.12		0.12	0.843				0.14	0.717		0.007	0.047				3.12	0.092	0.135	
Steal		0.408				0.001	0.98		0.000	0.946				0.71	0.410		0.03	0.760				0.27	0.609	0.013	

Legend. Data are means and standard deviations. INT: intervention group; CON: control group.

## Data Availability

The data presented in this study are available on request from the corresponding author. The data are not publicly available due to privacy reasons.
